# Early marginal peri-implant bone loss around tissue-level implants: a retrospective radiographic evaluation

**DOI:** 10.1186/s40729-025-00613-x

**Published:** 2025-03-12

**Authors:** A. Solderer, C. Giuliani, D. B. Wiedemeier, R. E. Jung, P. R. Schmidlin

**Affiliations:** 1 Clinic of Conservative and Preventive Dentistry, Division for Periodontology and Peri-Implant Diseases, Center for Dental Medicine, University of Zurich, Zurich, Switzerland; 2 Statistics Group, Center for Dental Medicine, University of Zurich, Zurich, Switzerland; 3 Clinic of Reconstructive Dentistry, Center for Dental Medicine, University of Zurich, Zurich, Switzerland

**Keywords:** Implant, Bone loss, Tissue level, Supracrestal tissue height, Retrospective

## Abstract

**Objectives:**

To retrospectively assess the potential impact of biological and host factors on radiographic bone loss following tissue-level implant placement and prosthetic rehabilitation.

**Methods:**

The University database was reviewed to identify patients treated with tissue-level implants between 2006 and 2020 at the University of Zurich, Switzerland. The study included patients who received screw-retained implant rehabilitations in the posterior area without simultaneous hard- or soft-tissue augmentations and had a follow-up period of at least 12 months. Radiographic measures of marginal bone loss and supracrestal tissue height were conducted using periapical x-rays at different time points. Additional factors analysed included age, gender, smoking status, history of periodontitis, jaw of treatment, type of reconstruction, and prosthetic emergence angle. Associations between marginal bone loss and potential explanatory variables were visualised and analysed. Elastic net regressions were applied to examine potential relationships with marginal bone loss.

**Results:**

A total of 1,479 patients were treated with tissue-level implants. After applying inclusion and exclusion criteria, 106 patients with 106 implants were included in the statistical evaluation after one year (T1, *n* = 106 implants), and 59 patients with 59 implants were evaluated after three years (T2, *n* = 59 implants). The mean marginal bone loss was 0.93 mm (SD 0.83) at T1 and 1.04 mm (SD 0.97) at T2. A strong correlation (Spearman) was found between mesial and distal bone loss. Smoking status and the jaw undergoing treatment were associated with bone loss. While these associations were observed in the univariate analysis, a more comprehensive multivariate analysis revealed that these variables had a limited effect on explaining radiographic bone loss.

**Conclusions:**

During the initial rehabilitation period in tissue-level implants in this cohort smoking status and jaw of treatment seemed to influence early peri-implant bone loss. Further, a strong correlation between mesial and distal MBL was observed. Additional research is required to determine factors contributing to early bone loss following implant-prosthetic rehabilitation.

## Graphical abstract

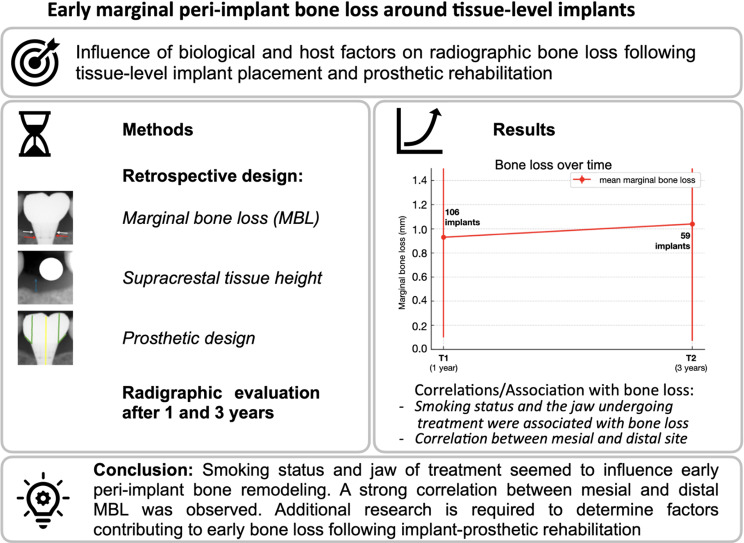

## Introduction

Peri-implant marginal bone stability is crucial for maintaining peri-implant health. To date, peri-implantitis remains the leading cause of implant complications and failure [1–3]. Peri-implantitis is strongly connected with progressive bone loss around implants [4]. According to the EFP/AAP consensus conference [4], it is defined as a pathological condition associated with peri-implant biofilm, characterised by increased probing depth compared to previous examinations, bone loss beyond initial crestal bone level changes, and the presence of bleeding on probing (BOP) or suppuration (SOP). Early marginal bone loss is a major risk factor for peri-implantitis [5]. Ravida´ et al. [6] highlighted the role of exposed implant threads after initial bone remodelling, noting an eight-fold higher odds ratio (OR) for developing peri-implantitis in implants with exposed threads compared to those without. Moreover, the risk increases four-fold with each additional exposed thread. Different factors have been identified as contributors to early marginal bone loss, also referred to as peri-implant bone remodelling [7].

Factors affecting early marginal bone loss (MBL) include surgical trauma [8], implant morphology [9], implant-abutment connection, prosthetic design [10], implant position [11], and soft tissue phenotype [12]. The formation of a biological peri-implant soft-tissue seal is essential to protect the crestal bone from the oral environment, necessitating an adequate peri-implant mucosal width to establish a proper epithelial-connective tissue attachment apparatus [13]. Meta-analyses suggest that implants with thicker soft tissue height (STH) experience approximately 0.5 mm less MBL during the first year compared to thinner phenotypes [12, 14, 15]. After three years, Tang et al. reported a smaller, non-significant difference of 0.2 mm [15]. Most MBL studies focus on bone level implants, with limited data available on tissue level implants. Spinato et al. examined bone remodelling in tissue-level implants five months post-surgery, excluding the prosthetic phase [16], and found STH substantially affected early bone loss. However, another study reported no significant effect of marginal tissue thickness when using tissue-level implants [17].

Although some clinicians consider tissue-level implants a more dated treatment approach compared to bone-level implants, evidence suggests that tissue-level implants have a significantly lower risk of peri-implantitis [9]. This may be attributed to the location of the implant-abutment micro-gaps near the bony crest in bone-level implants, which are prone to bacterial leakage, potentially leading to greater marginal bone loss [17]. This etiopathological connection underlines the need for a deeper understanding of the biological processes around tissue-level implants. Further, host factors such as smoking status and a history of periodontal disease appear to affect early bone remodelling. A recent radiographic study [18] over at least 36 months showed that light smokers experienced greater early bone loss than non-smokers, with the extent of bone loss increasing with daily cigarette consumption. Patients with a history of periodontitis had an almost 12-fold higher risk of developing more than 1 mm of MBL when presenting with thin supracrestal issue height (STH) [19]. Previous studies have also documented greater bone loss in individuals with a history of periodontal disease [20].

To the authors’ knowledge, outcomes regarding tissue-level implants remain scarce and controversial, leaving open the question of whether tissue-level implants are less susceptible to biological remodelling processes. This exploratory cohort study aimed to retrospectively evaluate the extent of radiographic bone loss following tissue-level implant placement and prosthetic rehabilitation. The analysis considered various biological and host factors, such as age, gender, smoking status, history of periodontitis, STH, jaw of treatment, type of reconstruction, and prosthetic emergence angle.

## Materials & methods

The current study adheres to the regulatory requirements of the Human Research Act and the Human Research Ordinance and was conducted the current version of the Declaration of Helsinki, ISO EN 14,155, as well as national legal and regulatory requirements. The investigation plan received approval from the Ethics Committee of the Canton of Zurich, Switzerland (ID: 2023 − 01820), and was registered in the German Register of Clinical Trials (DRKS00033665–20.02.2024 – www.drks.de). The study was carried out at the University of Zurich and conducted according to the STROBE guidelines. All included patients provided their informed consent by signing a general information and consent form.

### Study objectives

The objective of this study was to assess the mean marginal bone loss (MBL) around tissue-level implants one and three years after implant-prosthetic rehabilitation and to examine whether biological or host factors are associated with radiographic bone loss around tissue-level implants.

#### Primary outcome

The radiographically detectable amount of marginal bone loss at one year (T1) and three years (T2) after implant placement, compared to the marginal bone level at baseline (T0).

#### Secondary outcome

The influence of age, gender, smoking status, history of periodontitis, supracrestal tissue height, jaw of treatment, type of reconstruction, and prosthetic emergence profile on marginal bone loss at the different time points.

### Inclusion criteria

The following inclusion criteria were applied:

- Use of tissue-level implants (Straumann AG, Basel, Switzerland) placed between 2006 and 2020.
- Placement of tissue-level implants (Straumann AG, Basel, Switzerland) in posterior healed sites.
- Screw-retained prosthetics.
- Availability of X-rays taken before and at least 12 months after implant surgery.
- Patients aged ≥ 18 years.
- Trackable examination and anamnestic records.

### Exclusion criteria

Patients were excluded if any of the following criteria were present:

- Bone level implants.
- Immediate implant placement.
- Cemented restorations.
- History of radiation therapy in the head-neck area.
- Systemic or local diseases or conditions compromising healing or osseointegration (e.g., diabetes, osteoporosis).
- Use of medications affecting bone metabolism.
- Severe bruxism.
- Clear evidence of implant malpositioning or prosthetic misfit.
- Prosthetics with extending cantilevers.
- Pre-operative STH not measurable.
- Implants involving one- or two-staged bone augmentation.
- Implants with mucogingival surgery.
- Patients lacking regular supportive periodontal care.
- Documented refusal or withdrawal of consent.

#### Informed consent

was obtained from all included patients using the standard patient information form at the Center of Dental Medicine, University of Zurich.

**Fig. 1:**
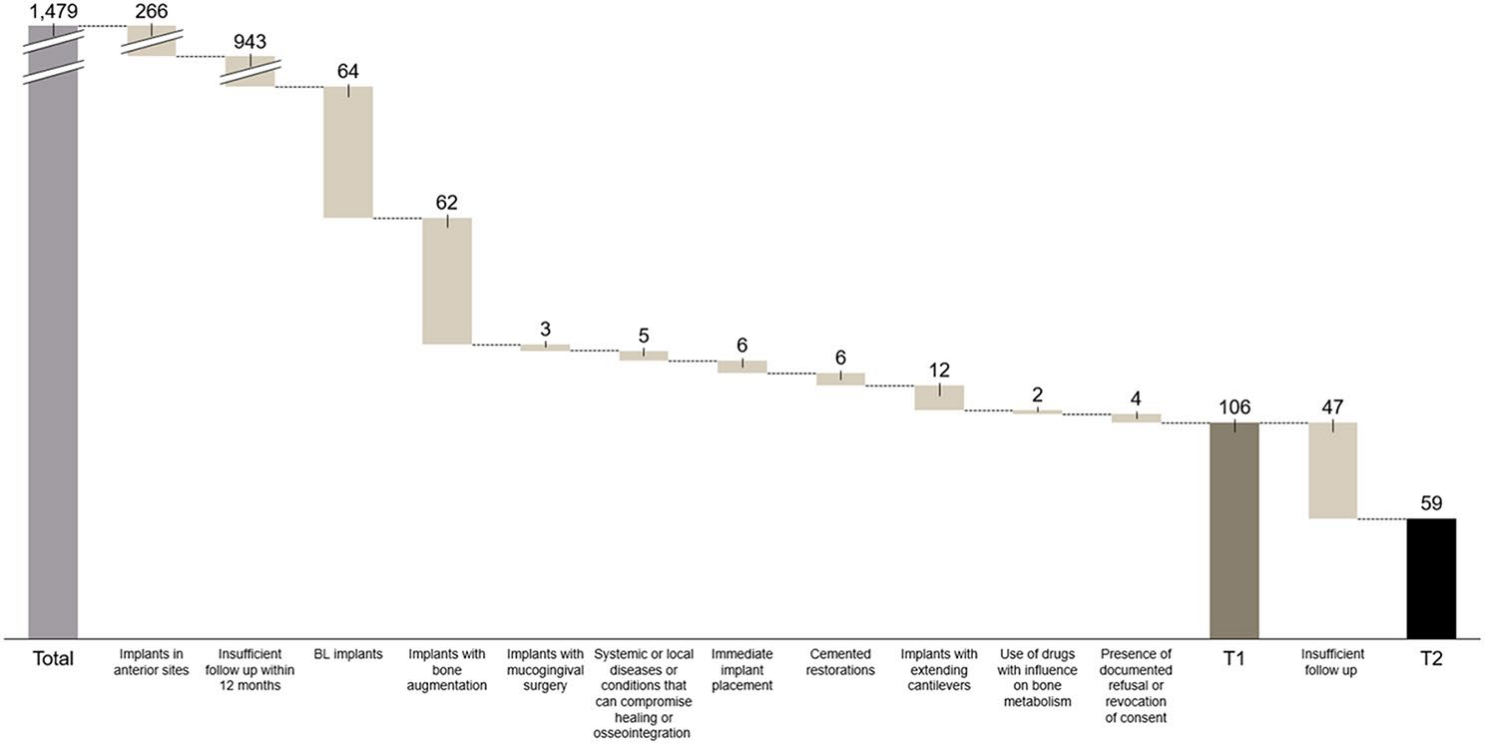
Flowchart illustrating patient inclusion and exclusion based on specific criteria

Figure 1 shows the flowchart of patients included for data analysis.

### Data assessment: patient data

The following patient data were extracted from medical records: age, general medical history, biological gender, smoking status, and history of periodontitis.

### **Data assessment: radiographic measurements**

**Fig. 2:**
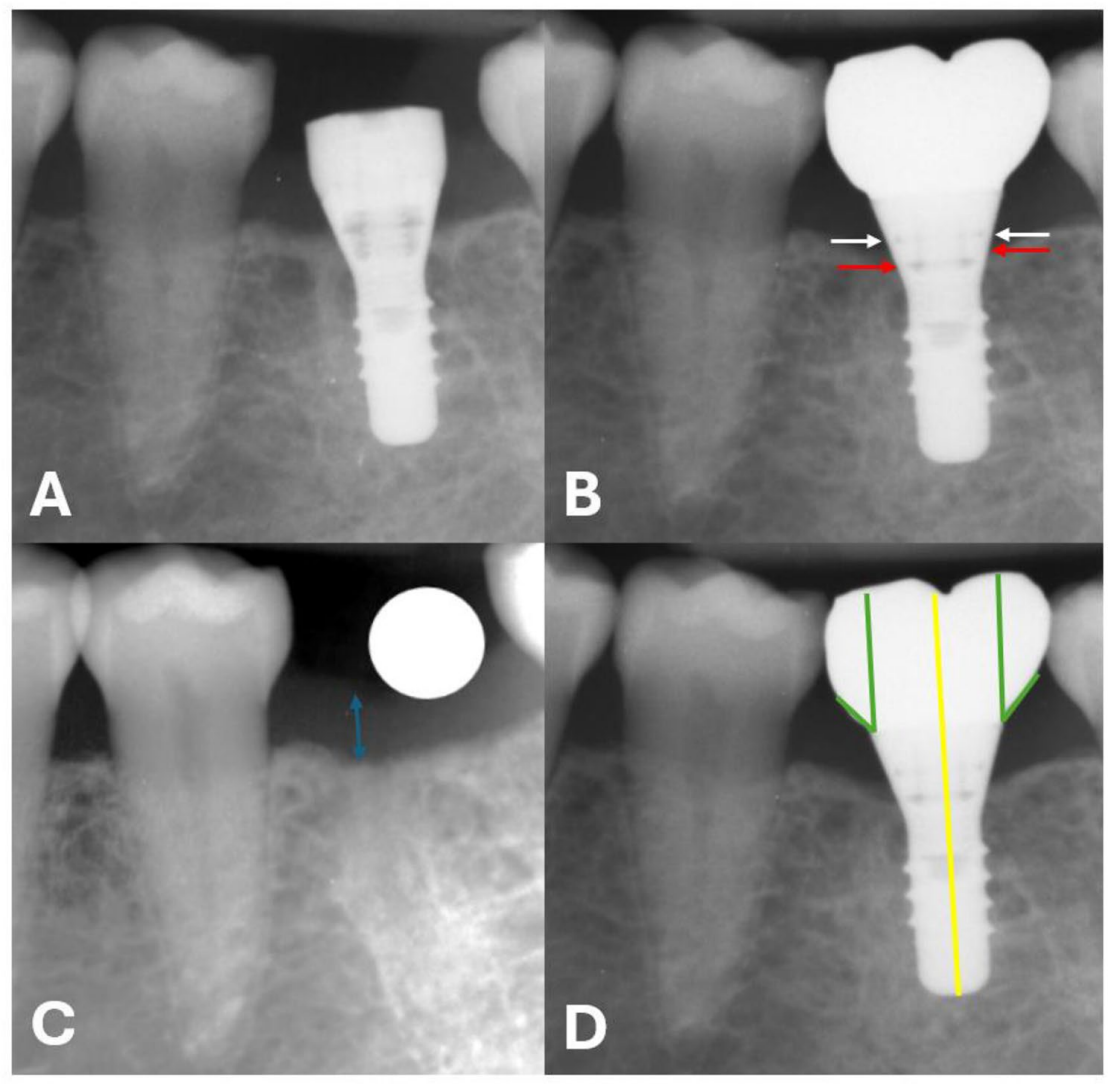
Outcome measurements. **A**: MBL at T0. **B**: MBL at T1-T2. **C**: STH at T0. **D**: Prosthetic emergence angle

Radiographic measurements were performed by two examiners (A.S. and C.G.) at a magnification of 100x using ImageJ software (Wayne Rasband, National Institutes of Health, Bethesda, MD, USA). Implant size was used to calibrate measurements in each radiograph (Fig. 2). The inter-examiner comparison showed a mean measurement error of 0.28 ± 0.23 mm. Any discrepancies were resolved through discussion until consensus was achieved.

The following measurements were conducted on periapical x-rays:1) Mesial and distal bone levels at implant placement (T0).

2) Mesial and distal bone levels one year (T1 = 12–16 months) and three years (T2 = 36–40 months) after implant surgery.

3) Supracrestal tissue height (STH) prior to implant placement (T0).

4) Mesial and distal emergence profile angles of the prosthetic reconstruction.

All measurements were organised into a tabular format (Excel 2024). The recorded data included: age, biological gender, smoking status, STH, type of reconstruction (bridge or crown), emergence angle, history of periodontitis, and jaw of treatment (mandible vs. maxilla).

### Statistical analysis

Descriptive statistics were calculated for all variables. Pairwise scatterplots and boxplots were used to visualise relationships. Spearman rank correlations with corresponding p-values were calculated to assess associations between target and continuous variables, while Wilcoxon rank-sum tests were used to evaluate associations between target and categorical variables. For MBL at T1 and T2, an elastic net model was fitted. The target variables were modelled individually as functions of the explanatory variables (age, gender, smoking status, history of periodontitis, supracrestal tissue height, jaw of treatment, type of reconstruction, and prosthetic emergence angle) and all possible pairwise interactions among these predictors up to the second degree. Both the target variables and STH were transformed using the hyperbolic sine function (IHS). Robustness during training was ensured through 5-fold cross-validation with five repeats and a random search for hyperparameter tuning (alpha and lambda), with a tuning length of 25. The `glmnet` package [21] was utilised for efficient variable selection and tuning. All analyses were conducted using the statistical software R [22] and the `GGally` package [23].

## Results

A total of 1,479 patients who received tissue-level implants between 2006 and 2020 were identified using in-house accounting software. Implants were placed and restored by several experienced practitioners, all adhering to clinic protocols within the clinics of the involved researchers.

Screenings were conducted independently by two authors (CG and AS). Initially, 132 implants in 106 patients were included. To ensure statistical independence, one implant per patient was randomly selected for 26 patients, resulting in a final sample of 106 implants from 106 patients at T1 and 59 patients with 59 implants at T2.

At T1, five implants exhibited more than 3 mm of bone loss, with two of these implants being lost before T2. These implant failures occurred due to biological complications (*n* = 1) and due to implant fracture (*n* = 1). At T2, two additional implants were removed because of progressive bone loss.

Table 1 shows an overview of the included patients and implants at one and three years.

**Table 1:** Baseline variables of patients included for T1 and T2 follow-up

At T1 (1 year)	*n*	%
**Gender**		
Male	49	46.2
Female	57	53.8
**Smoking**		
Yes	20	18.9
No	86	81.1
**History of Periodontitis**		
Yes	20	18.9
No	86	81.1
**Jaw of treatment**		
Mandible	66	62.3
Maxilla	40	37.7
**Prosthetic Design**		
Single Crown	99	93.4
Bridge	7	6.6
**Continous Variables**	**Mean**	**(SD)**
**Age (years)**	54.2	13.0
**Soft tissue height before surgery (mm)**	2.3	0.7
**At T2 (3 years)**		
**Gender**		
Male	37	62.7
Female	22	37.3
**Smoking**		
Yes	11	18.6
No	48	81.4
**History of Periodontitis**		
Yes	9	15.3
No	50	84.7
**Jaw of treatment**		
Mandible	38	64.4
Maxilla	21	35.6
**Prosthetic Design**		
Single Crown	57	96.6
Bridge	2	3.4
**Continous Variables at T2**	**Mean**	**(SD)**
**Age (years)**	53.9	12.5
**Soft tissue height before surgery (mm)**	2.4	0.7

**Table 2:** Descriptive statistics of marginal bone loss (mm) at different time points

Outcomes at T1	Mean (mm)	SD	Median (mm)	IQR
MBL mesial	0.91	0.89	0.77	1.32
MBL distal	0.95	0.87	0.84	1.17
MBL overall	0.93	0.83	0.79	0.96
**Outcomes at T2**				
MBL mesial	1.04	1.09	0.87	1.16
MBL distal	1.04	0.96	0.89	1.42
MBL overall	1.04	0.97	0.84	1.21

### Marginal bone loss

**Fig. 3:**
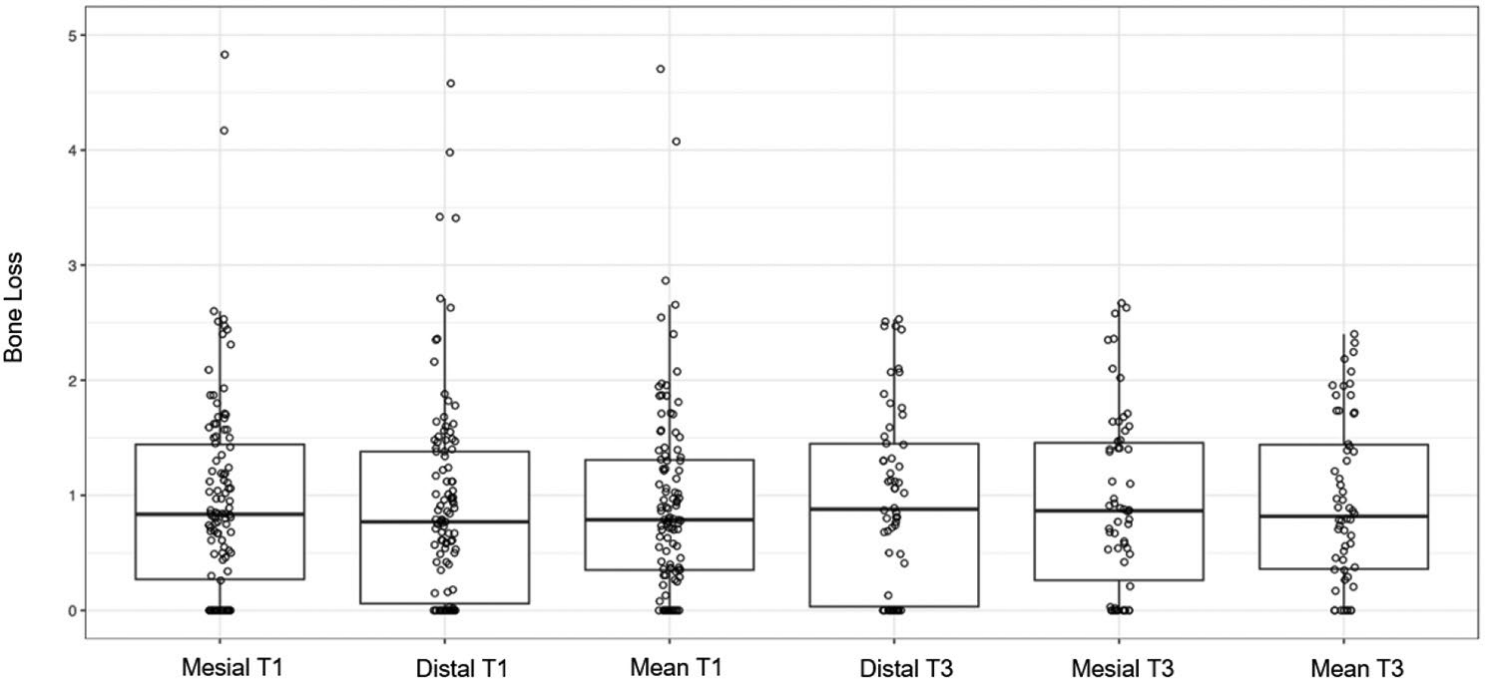
Marginal Bone Loss at T1 and T2

A mean marginal bone loss of 0.93 mm (SD: 0.83) was observed at T1, increasing to 1.04 mm (SD: 0.97) at T2. Detailed data are presented in Table 2 and Fig. 3.

### Correlations at T1

**Fig. 4:**
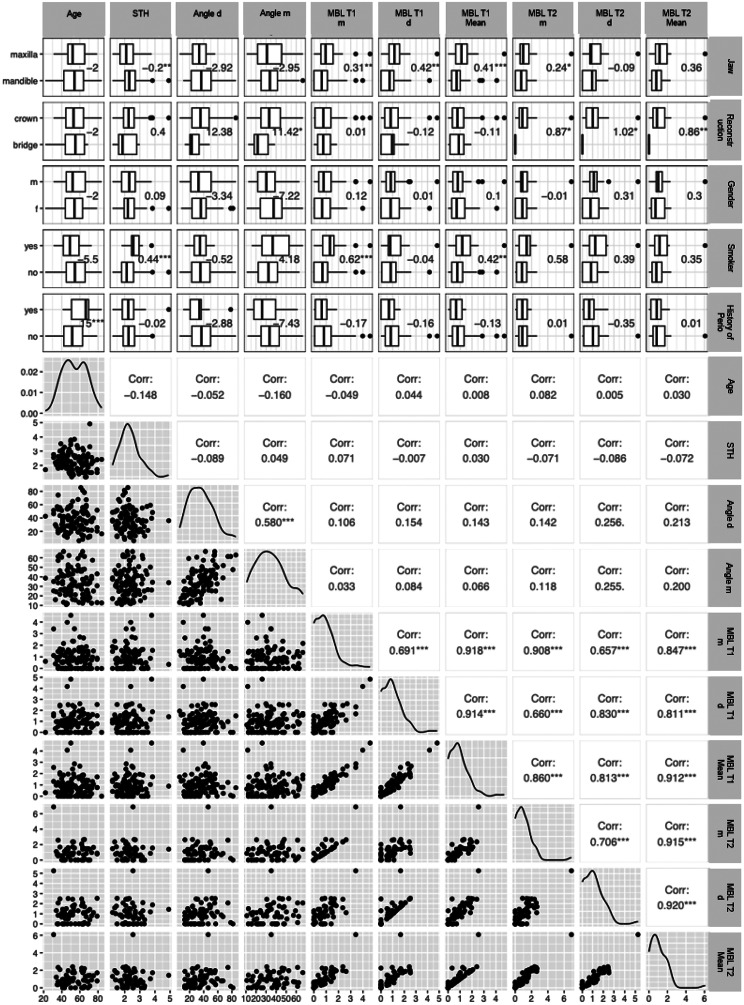
Pairwise relationships between the observed variables shown in box plots, scatter plots, including median differences and Spearman correlation coefficients

**Fig. 5:**
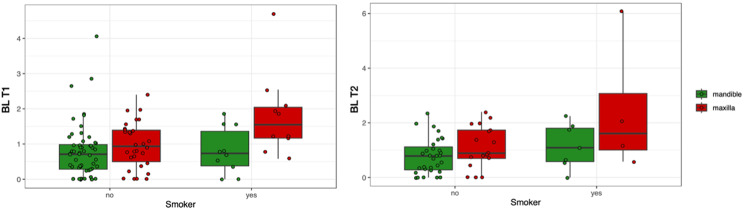
Effect of implant location and smoking status on mean bone loss (BL) at T1 and T2

Figure 4 illustrates the evaluation of relationships between the observed variables, showing a highly significant correlation between mesial and distal bone loss (0.918). Figures 4 and 5 also show a clear trend of higher marginal bone loss in implants placed in the upper jaw (*p* < 0.005) compared to the lower jaw and in smokers compared to non-smokers (*p* < 0.05). The combination of these two factors further amplifies the tendency for early marginal bone remodelling. No other factors showed meaningful relationships.

### Correlations at T2

Figure 4 illustrates the evaluation of potential influential factors, confirming a highly significant correlation between mesial and distal bone loss (0.706). Additionally, a strong correlation between MBL at T1 and T2 (0.912) was observed. The tendency for higher bone loss in the upper jaw and among smokers remained consistent up to T2, as illustrated in Fig. 5. The univariate analysis further indicated a significantly higher BL in crowns compared to bridges at T2 (*p* < 0.04), though including only limited observations.

### Multivariate modelling via elastic net

Careful model diagnostics revealed that none of the fitted models showed meaningful explanatory power, indicating that the selected explanatory variables did not significantly affect MBL at either T1 or T2. The R² values were 0.1, 0.16, and 0.16 for modelling distal, mesial, and average MBL at T1, respectively. Moreover, the root mean square error (RMSE) of the predictions was nearly identical to the standard deviation of the original data, further confirming the minimal impact of the explanatory variables on MBL. At T2, the coefficients of the explanatory variables were completely shrunk, reducing the predictions to the sample mean and showing no relationship between MBL and the selected variables.

## Discussion

This study provides a comprehensive analysis of early marginal bone loss around tissue-level implants, examining the potential effect of various biological and host factors. Strict inclusion criteria were applied, excluding any cases involving soft or hard tissue augmentation to minimise data bias. This rigorous selection process resulted in a final sample of 106 implants.

A mean marginal bone loss of 0.93 mm (SD 0.83) was observed after one year and 1.04 mm (SD 0.97) after three years. Notably, most implants experienced initial bone loss during the first year but remained stable without further bone loss by the three-year mark. These findings are consistent with results reported in similar studies [24]. However, other studies on Straumann TL implants have reported lower marginal bone loss, ranging from 0.1 to 0.2 mm after three years, based on larger patient cohorts [25, 26].

Evidence indicates that tissue-level implants have a lower risk of peri-implantitis compared to bone-level implants [9]. Studies conducted on animal models have reported that a more apical implant-abutment connection is associated with higher marginal bone loss, likely due to the closer proximity of the inflammation zone to the bone at the interface. Clinically, tissue-level implants with a supracrestal abutment connection show significantly fewer complications, further supporting their advantages in reducing peri-implantitis risk.

Although the comprehensive results of the multivariate machine learning approach did not show strong associations between the selected variables and MBL, some notable observations were made. Notably, mesial bone loss was frequently accompanied by distal bone loss, showing a strong relationship between the two.

These findings are consistent with previous studies. Schwarz et al. identified naturally occurring peri-implant bone defects, with circumferential bone defects being the most prevalent [27]. Similarly, a more recent study reported 2–3 wall defects involving both mesial and distal aspects in 55% of cases [28].

Second, a significant correlation was observed between bone loss at T1 and T2, showing that bone loss established within the first year after implant loading tends to persist over time.

After univariate statistical analysis, the explanatory variables found to be associated with bone loss after one or three years were the jaw of the implant location and smoking status. When combined, these factors resulted in even greater observed bone loss. These findings are consistent with recent studies, showing more pronounced MBL in smokers and in maxilla region [18, 19, 29].

No relevant relationship between the emergence angle and bone loss was identified in this cohort despite prior assumptions [30], that a wide emergence angle might affect early MBL around tissue-level implants. Substantial evidence shows that the prosthetic design of an implant restoration significantly affects peri-implant tissue health. Factors such as implant position, implant-abutment connection, emergence profile and angle, and material selection all affect bone remodelling and may contribute to marginal bone loss [31, 32], but these observations were taken predominantly in bone level implants. A recent meta-analysis with a follow-up of 4–10 years suggested that a prosthetic emergence angle of less than 30° is beneficial for maintaining marginal bone levels [10]. Strauss et al. [33] identified 40° as the cut-off for pronounced marginal bone loss. Herein the univariate analysis shows a significantly higher BL in crowns compared to bridges at T2. However, due to the very limited number of data points available at this time-point, the robustness of this finding is questionable. This result might be better described as an interesting pattern rather than a definitive conclusion.

In this study, no important relationship between STH and MBL was observed. These findings are consistent with a previous study conducted by van Eekeren [17], but contradict the findings by Spinato et al. [16], who reported greater early bone loss five months after surgery in patients with thin STH compared to those with thick STH. This observation is consistent with findings from studies on bone-level implants [12, 34]. However, it should be noted that Spinato et al. used a different type of tissue-level implant in their research [16].

The study findings highlight the complexity of factors affecting peri-implant bone loss, suggesting that variables not included in this study may play an important role or they might have a greater influence on bone around bone level implants. One potential reason for the lack of meaningful relationships between bone loss and the selected variables could be the relatively short follow-up period. Mombelli et al. [23] estimated that the prevalence of peri-implant inflammation with progressive bone loss ranges between 10% and 20% of implants over a 5–10 years follow-up period. This shows that most biological complications tend to occur after five years of loading, rather than in the short term, as examined in this study. Another possible explanation for early marginal bone remodelling is the slight subcrestal placement of the polished neck of tissue-level implants, potentially for esthetic purposes, which may have contributed to more pronounced remodelling. The retrospective nature of the study makes it challenging to fully exclude the possibility of submarginal implant placement, posing a limitation to the dataset. Additionally, the study has a few other limitations worth mentioning. First, MBL measurements relied on two-dimensional radiographs, which are limited to the interproximal areas and may not provide a complete representation of bone loss around the implant. While cone beam computed tomography (CBCT) scans offer a more accurate assessment of bone loss, their higher radiation exposure raises ethical concerns regarding their routine use. Additionally, the exploratory nature of the study, the relatively small sample size, and the limited follow-up duration may have affected the detection of more meaningful patterns between the selected variables and bone loss. Long-term follow-up was further challenged by patient drop-out. Moreover, factors such as surgical trauma, insertion torque, and bone density—which are known to affect early bone loss [31] —were not assessed in this retrospective radiographic study, presenting another limitation. In summary, the authors strongly encourage further research with larger patient cohorts and extended follow-up periods to identify potential baseline factors contributing to pronounced marginal bone loss and the eventual development of peri-implant disease.

## Conclusions

Of the selected explanatory variables smoking status and jaw of treatment showed univariate associations with bone loss within three years of rehabilitation in this patient cohort. A strong correlation between mesial and distal MBL was also observed. However, when comprehensively analysed in a multivariate fashion, the concerned variables had almost no explanatory power regarding bone loss. Additional research is required to identify the factors contributing to early bone loss around tissue-level implants.

## Data Availability

No datasets were generated or analysed during the current study.

## Abbreviations

BOP: Bleeding on Probing
MBL: Marginal Bone Loss
OR: Odds Ratio
SOP: Suppuration on Probing
STH: Soft Tissue Height
TL: Tissue Level
